# Di-μ_2_-isopropano­lato-octa­methyl­bis­(μ-4-methyl-5-sulfanyl­idene-4,5-dihydro-1*H*-1,2,4-triazol-1-ido-κ^2^ *N* ^1^:*N* ^2^)di-μ_3_-oxido-tetra­tin(IV)

**DOI:** 10.1107/S160053681204771X

**Published:** 2012-11-28

**Authors:** Ezzatollah Najafi, Mostafa M. Amini, Seik Weng Ng

**Affiliations:** a Department of Chemistry, General Campus, Shahid Beheshti University, Tehran 1983963113, Iran; b Department of Chemistry, University of Malaya, 50603 Kuala Lumpur, Malaysia

## Abstract

The tetra­nuclear title compound, [Sn_4_(CH_3_)_8_(C_3_H_7_O)_2_O_2_(C_3_H_4_N_3_S)_2_], lies about a center of inversion; the mol­ecule features a three-rung-staircase Sn_4_O_4_ core in which two Sn^IV^ atoms are bridged by the 4-methyl-5-sulfanyl­idene-4,5-dihydro-1*H*-1,2,4-triazol-1-ide group. The negatively charged N atom of the group binds to the terminal Sn^IV^ atom at a shorter distance [Sn—N = 2.236 (2) Å] compared with the neutral N atom that binds to the central Sn^IV^ atom [Sn← N = 2.805 (2) Å]. The terminal Sn^IV^ atom is five-coordinate in a *cis*-C_2_SnNO_2_ trigonal–bipyramidal geometry [C—Sn—C = 136.4 (1)°], whereas the central Sn^IV^ atom is six-coordinate in a C_2_SnNO_3_ skew-trazepoidal bipyramidal geometry [C—Sn—C = 145.4 (1)°]. The C atoms of the isopropoxy group are disordered over two positions in a 0.591 (7):0.409 (7) ratio.

## Related literature

For the [Sn_2_O(CH_3_)_4_(CH_3_O)(C_3_H_4_N_3_S)]_2_ homolog, see: Najafi *et al.* (2011 ▶).
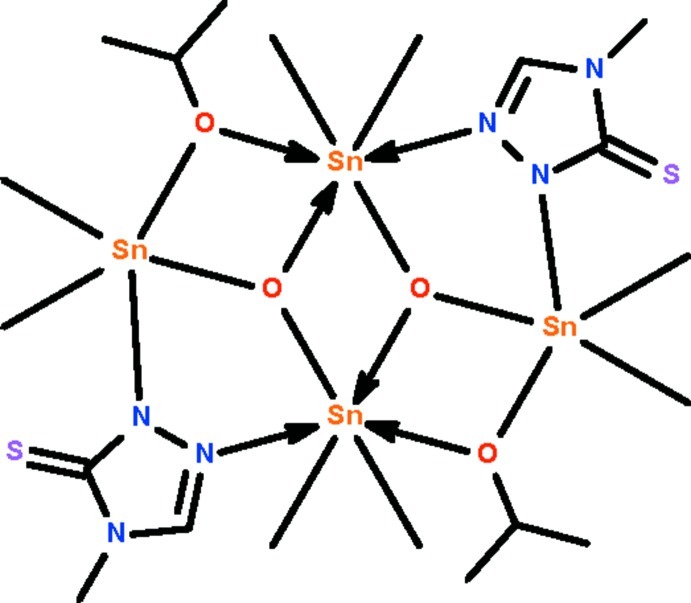


## Experimental

### 

#### Crystal data

[Sn_4_(CH_3_)_8_(C_3_H_7_O)_2_O_2_(C_3_H_4_N_3_S)_2_]
*M* *_r_* = 973.51
Monoclinic, 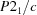

*a* = 9.6009 (4) Å
*b* = 10.0839 (4) Å
*c* = 18.3971 (6) Å
β = 94.667 (4)°
*V* = 1775.20 (12) Å^3^
*Z* = 2
Mo *K*α radiation
μ = 2.93 mm^−1^
*T* = 100 K
0.30 × 0.25 × 0.20 mm

#### Data collection

Agilent SuperNova (Dual, Cu at zero, Atlas) diffractometer
Absorption correction: multi-scan (*CrysAlis PRO*; Agilent, 2012) ▶ *T*_min_ = 0.474, *T*_max_ = 0.592
17413 measured reflections
4093 independent reflections
3613 reflections with *I* > 2σ(*I*)
*R*_int_ = 0.038

#### Refinement

*R*[*F* ^2^ > 2σ(*F* ^2^)] = 0.023
*wR*(*F* ^2^) = 0.052
*S* = 1.04
4093 reflections
174 parameters
21 restraints
H-atom parameters constrained
Δρ_max_ = 0.55 e Å^−3^
Δρ_min_ = −0.49 e Å^−3^

### 

Data collection: *CrysAlis PRO* (Agilent, 2012 ▶); cell refinement: *CrysAlis PRO*; data reduction: *CrysAlis PRO*; program(s) used to solve structure: *SHELXS97* (Sheldrick, 2008 ▶); program(s) used to refine structure: *SHELXL97* (Sheldrick, 2008 ▶); molecular graphics: *X-SEED* (Barbour, 2001 ▶); software used to prepare material for publication: *publCIF* (Westrip, 2010 ▶).

## Supplementary Material

Crystal structure: contains datablock(s) global, I. DOI: 10.1107/S160053681204771X/bt6864sup1.cif

Structure factors: contains datablock(s) I. DOI: 10.1107/S160053681204771X/bt6864Isup2.hkl

Additional supplementary materials:  crystallographic information; 3D view; checkCIF report

## Figures and Tables

**Fig. 1.**
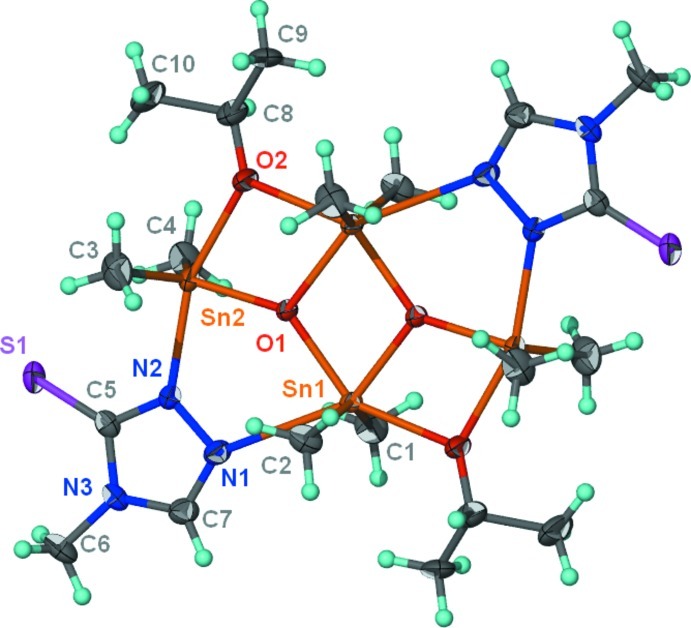
Thermal ellipsoid plot (Barbour, 2001) of Sn4O2(CH3)8(C3H7O)2(C3H4N3S)2 at the 70% probability level; hydrogen atoms are drawn as spheres of arbitrary radius. The disorder is not shown.

## supplementary crystallographic information

### Comment

The title compound (Scheme I, Fig. 1), a distannoxane, was the unexpected product from an attempt at synthesizing a dimethyltin 4-methyl-4*H*-1,2,4-triazol-3-thiolate that possesses a tin-sulfur linkage. In the reaction of diorganotin oxides with organic acids (particularly carboxylic acids), tetranuclear distannoxanes are sometimes formed; these compounds have four organic groups. In the present reaction, two of the four organic groups are replaced by ethoxide groups.

Tetranuclear Sn_4_O_2_(CH_3_)_8_(C_3_H_7_O)_2_(C_3_H_4_N_3_S)_2_ lies about a center-of-inversion; the molecule features a three-rung-staircase Sn_4_O_4_ core in which two Sn atoms are bridged by the C_3_H_4_N_3_S triazolate group. The negatively-charged N atom of the group binds to the terminal Sn atom at a shorter distance [Sn–N 2.236 (2) Å] compared with the neutral N atom that binds to the central Sn atom [Sn←N 2.805 (2) Å]. The terminal Sn atom is five-coordinate in a *cis*-C_3_SnNO trigonal bipyramid whereas the central Sn atom is six-coordinate in a C_2_SnNO_3_ skew-trazepoidal bipyramidal geometry.

### Experimental

Dimethyltin diisothiocyanate (1 mmol), 4-methyl-4*H*-1,2,4-triazole-3-thiol (1 mmol) and 1,10-phenanthroline (1 mmol) were loaded into a convection tube; several drops of triethylamine were added. The tube was filled with dry 2-propanol and kept at 333 K. Colorless crystals were collected from the side arm after several days.

### Refinement

Carbon-bound H-atoms were placed in calculated positions [C—H 0.95 to 0.98 Å, *U*_iso_(H) 1.2 to 1.5*U*_eq_(C)] and were included in the refinement in the riding model approximation.

The isopropoxy group is disordered over two positions in a 0.591 (7):0.409 ratio in respect of the carbon atoms. Pairs of 1,2-related distances were restrained to within 0.01 Å of each other, and the temperature factors of the primed atoms were set to those of the unprimed ones. The anisotropic temperatures were restrained to be nearly isotropic.

### Crystal data

**Table:**

[Sn_4_(CH_3_)_8_(C_3_H_7_O)_2_O_2_(C_3_H_4_N_3_S)_2_]	*F*(000) = 944
*M**_r_* = 973.51	*D*_x_ = 1.821 Mg m^−^^3^
Monoclinic, *P*2_1_/*c*	Mo *K*α radiation, λ = 0.71073 Å
Hall symbol: -P 2ybc	Cell parameters from 6879 reflections
*a* = 9.6009 (4) Å	θ = 3.1–27.5°
*b* = 10.0839 (4) Å	µ = 2.93 mm^−^^1^
*c* = 18.3971 (6) Å	*T* = 100 K
β = 94.667 (4)°	Prism, colorless
*V* = 1775.20 (12) Å^3^	0.30 × 0.25 × 0.20 mm
*Z* = 2	

### Data collection

**Table:**

Agilent SuperNova (Dual, Cu at zero, Atlas) diffractometer	4093 independent reflections
Radiation source: SuperNova (Mo) X-ray Source	3613 reflections with *I* > 2σ(*I*)
Mirror monochromator	*R*_int_ = 0.038
Detector resolution: 10.4041 pixels mm^-1^	θ_max_ = 27.6°, θ_min_ = 3.1°
ω scan	*h* = −12→12
Absorption correction: multi-scan (*CrysAlis PRO*; Agilent, 2012)	*k* = −13→12
*T*_min_ = 0.474, *T*_max_ = 0.592	*l* = −23→23
17413 measured reflections	

### Refinement

**Table:**

Refinement on *F*^2^	Primary atom site location: structure-invariant direct methods
Least-squares matrix: full	Secondary atom site location: difference Fourier map
*R*[*F*^2^ > 2σ(*F*^2^)] = 0.023	Hydrogen site location: inferred from neighbouring sites
*wR*(*F*^2^) = 0.052	H-atom parameters constrained
*S* = 1.04	*w* = 1/[σ^2^(*F*_o_^2^) + (0.0181*P*)^2^ + 1.4453*P*] where *P* = (*F*_o_^2^ + 2*F*_c_^2^)/3
4093 reflections	(Δ/σ)_max_ = 0.003
174 parameters	Δρ_max_ = 0.55 e Å^−^^3^
21 restraints	Δρ_min_ = −0.49 e Å^−^^3^

### Fractional atomic coordinates and isotropic or equivalent isotropic displacement parameters (Å^2^)

**Table:**

	*x*	*y*	*z*	*U*_iso_*/*U*_eq_	Occ. (<1)
Sn1	0.652613 (19)	0.422511 (18)	0.513713 (9)	0.01404 (6)	
Sn2	0.52915 (2)	0.709776 (19)	0.634092 (9)	0.01671 (6)	
S1	0.77775 (9)	0.79225 (8)	0.78514 (4)	0.02858 (18)	
O1	0.5301 (2)	0.57582 (19)	0.55360 (9)	0.0169 (4)	
O2	0.3284 (2)	0.7351 (2)	0.57053 (11)	0.0276 (5)	
N1	0.8106 (3)	0.5270 (2)	0.63540 (12)	0.0203 (5)	
N2	0.7409 (2)	0.6280 (2)	0.66839 (11)	0.0177 (5)	
N3	0.9416 (2)	0.6003 (2)	0.73020 (12)	0.0190 (5)	
C1	0.6250 (4)	0.2782 (3)	0.59418 (17)	0.0299 (7)	
H1A	0.6127	0.3221	0.6408	0.045*	
H1B	0.7074	0.2206	0.5995	0.045*	
H1C	0.5421	0.2248	0.5797	0.045*	
C2	0.7985 (3)	0.5418 (3)	0.46418 (15)	0.0246 (7)	
H2A	0.8123	0.6249	0.4914	0.037*	
H2B	0.7633	0.5614	0.4138	0.037*	
H2C	0.8877	0.4946	0.4643	0.037*	
C3	0.6090 (4)	0.8948 (3)	0.60284 (19)	0.0347 (8)	
H3A	0.5315	0.9526	0.5850	0.052*	
H3B	0.6718	0.8814	0.5641	0.052*	
H3C	0.6607	0.9363	0.6450	0.052*	
C4	0.4407 (3)	0.6321 (4)	0.72647 (15)	0.0320 (8)	
H4A	0.3414	0.6558	0.7245	0.048*	
H4B	0.4893	0.6692	0.7708	0.048*	
H4C	0.4503	0.5354	0.7270	0.048*	
C5	0.8202 (3)	0.6727 (3)	0.72657 (14)	0.0191 (6)	
C6	1.0595 (3)	0.6163 (4)	0.78449 (16)	0.0287 (7)	
H6A	1.1257	0.5433	0.7801	0.043*	
H6B	1.0259	0.6153	0.8334	0.043*	
H6C	1.1062	0.7009	0.7766	0.043*	
C7	0.9295 (3)	0.5128 (3)	0.67331 (15)	0.0231 (6)	
H7	0.9990	0.4500	0.6629	0.028*	
C8	0.2044 (6)	0.7853 (7)	0.5959 (3)	0.0377 (16)	0.591 (7)
H8	0.1592	0.7218	0.6285	0.045*	0.591 (7)
C9	0.1067 (12)	0.8255 (19)	0.5272 (8)	0.038 (3)	0.591 (7)
H9A	0.0934	0.7491	0.4944	0.058*	0.591 (7)
H9B	0.1497	0.8983	0.5017	0.058*	0.591 (7)
H9C	0.0161	0.8542	0.5424	0.058*	0.591 (7)
C10	0.242 (2)	0.9267 (11)	0.6349 (6)	0.049 (3)	0.591 (7)
H10A	0.3190	0.9148	0.6726	0.074*	0.591 (7)
H10B	0.1597	0.9605	0.6572	0.074*	0.591 (7)
H10C	0.2697	0.9901	0.5984	0.074*	0.591 (7)
C8'	0.2376 (9)	0.8453 (9)	0.5658 (5)	0.0377 (16)	0.41
H8'	0.2723	0.9185	0.5353	0.045*	0.409 (7)
C9'	0.0850 (17)	0.802 (3)	0.5403 (13)	0.038 (3)	0.41
H9D	0.0874	0.7225	0.5100	0.058*	0.409 (7)
H9E	0.0381	0.8740	0.5119	0.058*	0.409 (7)
H9F	0.0337	0.7833	0.5830	0.058*	0.409 (7)
C10'	0.222 (3)	0.8905 (19)	0.6497 (8)	0.049 (3)	0.41
H10D	0.3053	0.8620	0.6804	0.074*	0.409 (7)
H10E	0.1390	0.8495	0.6673	0.074*	0.409 (7)
H10F	0.2140	0.9872	0.6520	0.074*	0.409 (7)

### Atomic displacement parameters (Å^2^)

**Table:**

	*U*^11^	*U*^22^	*U*^33^	*U*^12^	*U*^13^	*U*^23^
Sn1	0.01409 (10)	0.01444 (11)	0.01334 (9)	0.00295 (7)	−0.00045 (7)	−0.00018 (7)
Sn2	0.01739 (10)	0.01539 (11)	0.01707 (10)	0.00134 (7)	−0.00036 (8)	−0.00515 (7)
S1	0.0253 (4)	0.0286 (5)	0.0312 (4)	−0.0025 (3)	−0.0012 (3)	−0.0154 (3)
O1	0.0166 (10)	0.0185 (11)	0.0149 (9)	0.0055 (8)	−0.0037 (8)	−0.0056 (7)
O2	0.0232 (11)	0.0292 (13)	0.0288 (11)	0.0121 (10)	−0.0074 (9)	−0.0140 (9)
N1	0.0213 (13)	0.0218 (14)	0.0179 (11)	0.0058 (10)	0.0015 (10)	−0.0026 (10)
N2	0.0180 (12)	0.0163 (13)	0.0185 (11)	0.0013 (10)	−0.0003 (10)	−0.0041 (9)
N3	0.0151 (12)	0.0247 (14)	0.0167 (11)	−0.0040 (10)	−0.0009 (9)	0.0004 (9)
C1	0.0340 (18)	0.0247 (18)	0.0297 (16)	−0.0028 (14)	−0.0043 (14)	0.0093 (13)
C2	0.0203 (15)	0.0321 (19)	0.0217 (14)	−0.0023 (13)	0.0037 (12)	0.0051 (13)
C3	0.041 (2)	0.0187 (18)	0.0428 (19)	−0.0071 (15)	−0.0072 (16)	0.0056 (14)
C4	0.0277 (17)	0.048 (2)	0.0213 (15)	−0.0071 (16)	0.0084 (13)	−0.0066 (14)
C5	0.0172 (14)	0.0211 (16)	0.0188 (13)	−0.0040 (12)	0.0005 (11)	0.0005 (11)
C6	0.0181 (15)	0.039 (2)	0.0281 (15)	−0.0024 (14)	−0.0070 (13)	0.0017 (14)
C7	0.0211 (15)	0.0291 (18)	0.0195 (13)	0.0050 (13)	0.0029 (12)	−0.0025 (12)
C8	0.029 (3)	0.041 (4)	0.042 (4)	0.015 (3)	−0.006 (2)	−0.018 (2)
C9	0.024 (4)	0.034 (6)	0.056 (5)	0.016 (3)	−0.004 (4)	−0.009 (4)
C10	0.045 (5)	0.039 (7)	0.063 (5)	0.021 (5)	−0.009 (4)	−0.030 (5)
C8'	0.029 (3)	0.041 (4)	0.042 (4)	0.015 (3)	−0.006 (2)	−0.018 (2)
C9'	0.024 (4)	0.034 (6)	0.056 (5)	0.016 (3)	−0.004 (4)	−0.009 (4)
C10'	0.045 (5)	0.039 (7)	0.063 (5)	0.021 (5)	−0.009 (4)	−0.030 (5)

### Geometric parameters (Å, º)

**Table:**

Sn1—O1^i^	2.0633 (18)	C3—H3A	0.9800
Sn1—C1	2.108 (3)	C3—H3B	0.9800
Sn1—C2	2.109 (3)	C3—H3C	0.9800
Sn1—O1	2.1101 (18)	C4—H4A	0.9800
Sn1—O2^i^	2.2377 (19)	C4—H4B	0.9800
Sn1—N1	2.805 (2)	C4—H4C	0.9800
Sn2—O1	2.0050 (17)	C6—H6A	0.9800
Sn2—C4	2.111 (3)	C6—H6B	0.9800
Sn2—C3	2.115 (3)	C6—H6C	0.9800
Sn2—O2	2.187 (2)	C7—H7	0.9500
Sn2—N2	2.236 (2)	C8—C9	1.566 (9)
S1—C5	1.689 (3)	C8—C10	1.623 (9)
O1—Sn1^i^	2.0633 (18)	C8—H8	1.0000
O2—C8	1.408 (5)	C9—H9A	0.9800
O2—C8'	1.411 (7)	C9—H9B	0.9800
O2—Sn1^i^	2.2377 (19)	C9—H9C	0.9800
N1—C7	1.296 (4)	C10—H10A	0.9800
N1—N2	1.386 (3)	C10—H10B	0.9800
N2—C5	1.340 (3)	C10—H10C	0.9800
N3—C7	1.367 (4)	C8'—C9'	1.563 (11)
N3—C5	1.372 (4)	C8'—C10'	1.629 (10)
N3—C6	1.456 (3)	C8'—H8'	1.0000
C1—H1A	0.9800	C9'—H9D	0.9800
C1—H1B	0.9800	C9'—H9E	0.9800
C1—H1C	0.9800	C9'—H9F	0.9800
C2—H2A	0.9800	C10'—H10D	0.9800
C2—H2B	0.9800	C10'—H10E	0.9800
C2—H2C	0.9800	C10'—H10F	0.9800
			
O1^i^—Sn1—C1	106.13 (11)	Sn2—C3—H3C	109.5
O1^i^—Sn1—C2	107.34 (10)	H3A—C3—H3C	109.5
C1—Sn1—C2	145.41 (12)	H3B—C3—H3C	109.5
O1^i^—Sn1—O1	74.45 (8)	Sn2—C4—H4A	109.5
C1—Sn1—O1	98.98 (11)	Sn2—C4—H4B	109.5
C2—Sn1—O1	98.10 (10)	H4A—C4—H4B	109.5
O1^i^—Sn1—O2^i^	72.74 (7)	Sn2—C4—H4C	109.5
C1—Sn1—O2^i^	91.03 (11)	H4A—C4—H4C	109.5
C2—Sn1—O2^i^	90.66 (11)	H4B—C4—H4C	109.5
O1—Sn1—O2^i^	147.18 (7)	N2—C5—N3	106.6 (2)
O1^i^—Sn1—N1	148.65 (7)	N2—C5—S1	126.7 (2)
C1—Sn1—N1	77.85 (10)	N3—C5—S1	126.6 (2)
C2—Sn1—N1	78.21 (9)	N3—C6—H6A	109.5
O1—Sn1—N1	74.22 (7)	N3—C6—H6B	109.5
O2^i^—Sn1—N1	138.60 (7)	H6A—C6—H6B	109.5
O1—Sn2—C4	111.77 (11)	N3—C6—H6C	109.5
O1—Sn2—C3	111.73 (11)	H6A—C6—H6C	109.5
C4—Sn2—C3	136.43 (14)	H6B—C6—H6C	109.5
O1—Sn2—O2	74.96 (7)	N1—C7—N3	110.9 (3)
C4—Sn2—O2	94.53 (11)	N1—C7—H7	124.5
C3—Sn2—O2	94.29 (11)	N3—C7—H7	124.5
O1—Sn2—N2	84.34 (8)	O2—C8—C9	106.9 (8)
C4—Sn2—N2	92.98 (11)	O2—C8—C10	107.4 (8)
C3—Sn2—N2	93.49 (12)	C9—C8—C10	103.1 (10)
O2—Sn2—N2	159.29 (8)	O2—C8—H8	112.9
Sn2—O1—Sn1^i^	112.53 (9)	C9—C8—H8	112.9
Sn2—O1—Sn1	141.87 (9)	C10—C8—H8	112.9
Sn1^i^—O1—Sn1	105.55 (8)	C8—C9—H9A	109.5
C8—O2—Sn2	126.6 (3)	C8—C9—H9B	109.5
C8'—O2—Sn2	129.7 (4)	H9A—C9—H9B	109.5
C8—O2—Sn1^i^	127.2 (3)	C8—C9—H9C	109.5
C8'—O2—Sn1^i^	126.9 (4)	H9A—C9—H9C	109.5
Sn2—O2—Sn1^i^	99.75 (8)	H9B—C9—H9C	109.5
C7—N1—N2	106.3 (2)	C8—C10—H10A	109.5
C7—N1—Sn1	141.58 (19)	C8—C10—H10B	109.5
N2—N1—Sn1	112.10 (15)	H10A—C10—H10B	109.5
C5—N2—N1	109.5 (2)	C8—C10—H10C	109.5
C5—N2—Sn2	123.10 (19)	H10A—C10—H10C	109.5
N1—N2—Sn2	127.45 (16)	H10B—C10—H10C	109.5
C7—N3—C5	106.7 (2)	O2—C8'—C9'	111.1 (13)
C7—N3—C6	127.5 (3)	O2—C8'—C10'	105.3 (11)
C5—N3—C6	125.8 (2)	C9'—C8'—C10'	102.0 (16)
Sn1—C1—H1A	109.5	O2—C8'—H8'	112.6
Sn1—C1—H1B	109.5	C9'—C8'—H8'	112.6
H1A—C1—H1B	109.5	C10'—C8'—H8'	112.6
Sn1—C1—H1C	109.5	C8'—C9'—H9D	109.5
H1A—C1—H1C	109.5	C8'—C9'—H9E	109.5
H1B—C1—H1C	109.5	H9D—C9'—H9E	109.5
Sn1—C2—H2A	109.5	C8'—C9'—H9F	109.5
Sn1—C2—H2B	109.5	H9D—C9'—H9F	109.5
H2A—C2—H2B	109.5	H9E—C9'—H9F	109.5
Sn1—C2—H2C	109.5	C8'—C10'—H10D	109.5
H2A—C2—H2C	109.5	C8'—C10'—H10E	109.5
H2B—C2—H2C	109.5	H10D—C10'—H10E	109.5
Sn2—C3—H3A	109.5	C8'—C10'—H10F	109.5
Sn2—C3—H3B	109.5	H10D—C10'—H10F	109.5
H3A—C3—H3B	109.5	H10E—C10'—H10F	109.5
			
C4—Sn2—O1—Sn1^i^	87.73 (13)	O1—Sn1—N1—N2	1.24 (17)
C3—Sn2—O1—Sn1^i^	−89.81 (13)	O2^i^—Sn1—N1—N2	−179.37 (16)
O2—Sn2—O1—Sn1^i^	−1.17 (9)	C7—N1—N2—C5	−0.3 (3)
N2—Sn2—O1—Sn1^i^	178.69 (11)	Sn1—N1—N2—C5	179.15 (17)
C4—Sn2—O1—Sn1	−88.87 (19)	C7—N1—N2—Sn2	179.8 (2)
C3—Sn2—O1—Sn1	93.60 (19)	Sn1—N1—N2—Sn2	−0.7 (3)
O2—Sn2—O1—Sn1	−177.76 (18)	O1—Sn2—N2—C5	179.9 (2)
N2—Sn2—O1—Sn1	2.09 (16)	C4—Sn2—N2—C5	−68.5 (2)
O1^i^—Sn1—O1—Sn2	176.7 (2)	C3—Sn2—N2—C5	68.4 (2)
C1—Sn1—O1—Sn2	72.38 (18)	O2—Sn2—N2—C5	−179.7 (2)
C2—Sn1—O1—Sn2	−77.43 (18)	O1—Sn2—N2—N1	−0.2 (2)
O2^i^—Sn1—O1—Sn2	178.55 (13)	C4—Sn2—N2—N1	111.4 (2)
N1—Sn1—O1—Sn2	−2.19 (15)	C3—Sn2—N2—N1	−111.8 (2)
O1^i^—Sn1—O1—Sn1^i^	0.0	O2—Sn2—N2—N1	0.2 (4)
C1—Sn1—O1—Sn1^i^	−104.36 (11)	N1—N2—C5—N3	0.5 (3)
C2—Sn1—O1—Sn1^i^	105.84 (11)	Sn2—N2—C5—N3	−179.64 (17)
O2^i^—Sn1—O1—Sn1^i^	1.82 (19)	N1—N2—C5—S1	−178.2 (2)
N1—Sn1—O1—Sn1^i^	−178.92 (10)	Sn2—N2—C5—S1	1.6 (4)
O1—Sn2—O2—C8	154.1 (4)	C7—N3—C5—N2	−0.5 (3)
C4—Sn2—O2—C8	42.7 (4)	C6—N3—C5—N2	179.2 (3)
C3—Sn2—O2—C8	−94.6 (4)	C7—N3—C5—S1	178.3 (2)
N2—Sn2—O2—C8	153.7 (4)	C6—N3—C5—S1	−2.1 (4)
O1—Sn2—O2—C8'	−158.3 (6)	N2—N1—C7—N3	0.0 (3)
C4—Sn2—O2—C8'	90.3 (6)	Sn1—N1—C7—N3	−179.2 (2)
C3—Sn2—O2—C8'	−47.0 (6)	C5—N3—C7—N1	0.3 (3)
N2—Sn2—O2—C8'	−158.7 (6)	C6—N3—C7—N1	−179.4 (3)
O1—Sn2—O2—Sn1^i^	1.01 (8)	C8'—O2—C8—C9	52.9 (10)
C4—Sn2—O2—Sn1^i^	−110.34 (12)	Sn2—O2—C8—C9	162.6 (6)
C3—Sn2—O2—Sn1^i^	112.37 (12)	Sn1^i^—O2—C8—C9	−51.5 (9)
N2—Sn2—O2—Sn1^i^	0.6 (3)	C8'—O2—C8—C10	−57.2 (10)
O1^i^—Sn1—N1—C7	178.4 (3)	Sn2—O2—C8—C10	52.5 (8)
C1—Sn1—N1—C7	77.3 (3)	Sn1^i^—O2—C8—C10	−161.6 (5)
C2—Sn1—N1—C7	−77.5 (3)	C8—O2—C8'—C9'	−56.5 (13)
O1—Sn1—N1—C7	−179.6 (3)	Sn2—O2—C8'—C9'	−157.3 (11)
O2^i^—Sn1—N1—C7	−0.2 (4)	Sn1^i^—O2—C8'—C9'	48.5 (14)
O1^i^—Sn1—N1—N2	−0.8 (3)	C8—O2—C8'—C10'	53.2 (12)
C1—Sn1—N1—N2	−101.9 (2)	Sn2—O2—C8'—C10'	−47.6 (13)
C2—Sn1—N1—N2	103.28 (19)	Sn1^i^—O2—C8'—C10'	158.2 (10)

Symmetry code: (i) −*x*+1, −*y*+1, −*z*+1.
